# LC-PDA-MS and GC-MS Analysis of *Scorzonera hispanica* Seeds and Their Effects on Human Breast Cancer Cell Lines

**DOI:** 10.3390/ijms231911584

**Published:** 2022-09-30

**Authors:** Karolina Lendzion, Agnieszka Gornowicz, Jakub W. Strawa, Katarzyna Bielawska, Robert Czarnomysy, Bożena Popławska, Krzysztof Bielawski, Michał Tomczyk, Wojciech Miltyk, Anna Bielawska

**Affiliations:** 1 Department of Biotechnology, Faculty of Pharmacy, Medical University of Bialystok, ul. Kilińskiego 1, 15-089 Bialystok, Poland; 2 Department of Pharmacognosy, Faculty of Pharmacy, Medical University of Bialystok, ul. Mickiewicza 2A, 15-230 Bialystok, Poland; 3 Department of Pharmaceutical and Biopharmaceutical Analysis, Faculty of Pharmacy, Medical University of Bialystok, ul. Mickiewicza 2D, 15-222 Bialystok, Poland; 4 Department of Synthesis and Technology of Drugs, Faculty of Pharmacy, Medical University of Bialystok, ul. Kilińskiego 1, 15-089 Bialystok, Poland

**Keywords:** *Scorzonera*, seeds, polyphenols, LC-PDA-MS, GC-MS, breast cancer, biological activity

## Abstract

*Scorzonera hispanica* is an herbaceous perennial cultivated in Central and Southern Europe. This study aimed to qualitatively and quantitatively evaluate the composition of oil, extracts, and fractions (**SH1-SH12**) obtained from *S. hispanica* seeds. Furthermore, an evaluation of biological activities in breast cancer cell lines was also performed. GC-MS analysis revealed that the primary components of the seed oil (**SH12**) were fatty acids and β-sitosterol. In the evaluation of extracts (**SH1**-**SH3**, **SH8**-**SH10**) and fractions (**SH4**-**SH7**, **SH11**) composition, the presence of apigenin, derivatives of *p*-coumaric and caffeic acids, was reported. In the biological assays, methanolic extract (**SH1**), diethyl ether (**SH4**), and chloroform (**SH11**) fractions exhibited cytotoxicity toward cells. The highest activity was observed for fatty acids- and 3,4-dimethoxycinnamate-rich **SH11** (IC_50_: 399.18 μg/mL for MCF-7, 781.26 μg/mL for MDA-MB-231). **SH11** was also observed to induce apoptosis in MCF-7 cells (52.4%). **SH1**, **SH4**, and **SH11** attenuate signaling pathways and affect the expression of apoptosis-, autophagy-, and inflammation-related proteins. **SH12** was non-toxic toward either cancer or normal cell lines in concentrations up to 1 mg/mL. The results suggest that *S. hispanica* seeds exhibit a wide range of potential uses as a source of oil and bioactive compounds for complementary therapy of breast cancer.

## 1. Introduction

*Scorzonera* L. (Asteraceae) is a genus comprising approximately 200 plants, growing across Europe, Asia, and northern Africa [1,2]. In desert regions, some *Scorzonera* species are used as forage [3]. An species endemic to Central Asia, *S. tau-saghyz* Lipsch. and Bosse, is cultivated as a rubber-bearing plant [4]. In traditional medicine, plants of the genus *Scorzonera* play a particular role, including their antidiabetic, analgesic, or antipyretic activities [5,6,7,8]. *Scorzonera* species have also been a subject of interest in terms of their content of bioactive compounds [9]. The cytotoxic [10,11], anti-inflammatory [12,13,14], and wound healing [15,16] activities of extracts from *Scorzonera* species, in addition to isolated compounds, were evaluated in multiple in vitro and in vivo studies.

*Scorzonera hispanica* L. (black salsify) syn.: *Pseudopodospermum hispanicum* (L.) Zaika, Sukhor. and N. Kilian (Asteraceae) is a perennial plant, spread across Europe and southern Siberia [17]. In the traditional medicine of Europe, *S. hispanica* roots were used to treat colds, stimulate appetite, and as a mucolytic agent in lung diseases [8,18]. In modern times, black salsify root is a valued vegetable. Previous studies on the species have indicated that aerial parts of the plant contain flavonoids, in addition to caffeic acid and its derivatives [17,19]. In the aerial parts, the presence of lignans, sesquiterpenoids, caffeic acid derivatives, and inulin was reported [17,18,20]. (−)-Syringaresinol, isolated from the roots of black salsify [17], was previously observed to exhibit cytotoxicity to several carcinoma cell lines, including breast cancer [21,22]. No previous reports on the composition or biological activity of the seeds of *S. hispanica* are available in the literature. To our best knowledge, this is the first attempt to evaluate the phytochemical profile and bioactivity of these products obtained from *S. hispanica* seeds.

The aim of this study was to obtain and elucidate the components of oil, extracts, and fractions obtained from the seeds of *S. hispanica* and their activities against two human mammary carcinoma cell lines in addition to normal cells (human skin fibroblasts). The GC-MS analysis and cytotoxicity assessment of the oil were aimed to evaluate the seeds as a novel plant oil source. Six extracts and five fractions using various methods were obtained and their phytochemical profiles using LC-PDA-MS and GC-MS techniques, in addition to their influence on viability and DNA biosynthesis in the mentioned cell lines, were evaluated. The effect of the three most promising products on apoptosis induction in the MCF-7 cell line was assessed. Then, the expression of apoptosis- and autophagy-related proteins using the Western blot technique was investigated. The influence of selected extracts on the concentration of proteins participating in cell signaling pathways and their anti-inflammatory potential was also assessed. As the anticancer activity of *S. hispanica* seeds is yet to be elucidated, we investigated their effect on the concentration of phosphorylated extracellular signal-regulated kinases 1 and 2 (ERK 1/2), in addition to phosphorylated protein kinase B (p-Akt), as previous clinical studies have indicated a correlation between the expression of those two proteins in breast cancer patients. Coexpression of p-Akt and p-Erk 1/2 was reported as a potential predictor of a reduced disease-free survival time for patients diagnosed in the early stage of breast cancer [23]. Therefore, inhibition of those two proteins involved in cell signaling pathways leads to cell death and is the desired effect of anticancer agents. As focal adhesion kinase (FAK) is involved in cell migration, adhesion, and apoptosis, and regulates PI3K/Akt cell signaling pathway [24,25], we assessed the influence of **SH1**, **SH4**, and **SH11** on the expression of phosphorylated FAK (p-FAK) in MCF-7 breast cancer cells. Finally, ERK 1/2 and Akt both lead to the inhibition of the expression of pro-apoptotic Bad protein which inhibits the activity of anti-apoptotic BCL-2. BCL-2 in turn blocks the expression of Bax [26]. Additionally, BCL-2 prevents Beclin-1 from initiating the process of autophagy [27]. Apoptosis and autophagy often occur simultaneously in the cell [28]. Hence, to investigate the influence of the assessed extract and fractions on autophagy in breast cancer cells, we evaluated the expression of ATG5 and LC3B proteins. In addition to the apoptosis-autophagy investigation, we assessed the influence of **SH1**, **SH4**, and **SH11** on pro- (IL-8, TNF-α) and anti-inflammatory (IL-10) cytokines. As IL-8 and TNF-α are associated with cancer progression and metastasis [29,30], the inhibitory effect of the extracts on those cytokines was anticipated. Interleukin-10, which is generally considered to possess anti-inflammatory properties, plays a dual role in breast cancer. It can exert both pro-tumor and anti-tumor activity [31,32]. Therefore, we investigated how **SH1**, **SH4**, and **SH11** affect the concentration of IL-10 in MCF-7 cells.

## 2. Results

### 2.1. GC-MS Analysis of SH1, SH9-SH12

The GC-MS analysis of **SH1**, **SH9-SH11** revealed that the dominating groups of compounds for **SH1** were carbohydrates (54.6% relative content; with sucrose as the main constituent) and organic polyols (15.8%; main constituent: D-chiro-inositol). Another interesting group present in **SH1** was phenolic compounds, with caffeic acid as the primary phenolic acid detected in the sample. The presence of quinic acid was also reported in **SH1**. For **SH9** and **SH12**, 41.7% and 62.2% of the relative composition were fatty acids, with most being linoleic acid. Fatty acid esters, with butyl 9,12-octadecadienoate and conjugated linoleic acid esters, were 18.8% of relative extract **SH9** composition. Noteworthily, **SH9** and **SH12** were observed to contain a notable amount of phytosterols like β-sitosterol, 5α-stigmast-7-en-3β-ol, and stigmasterol. **SH10** relatively consisted of 44.2% fatty acids, with linoleic acid (LA), oleic acid (OA), and palmitic acid (PA) as the primary fatty acids. Glycerol was 15.7% of the total phytochemicals detected in **SH10**. **SH11** relatively consisted of 33% fatty acids (linoleic acid, conjugated linoleic acid, oleic, and palmitic acids) and 21.16% methyl 3,4-dimethoxycinnamate. The primary components of **SH12** were fatty acids (61.8%; including 27.2% linoleic acid) and phytosterols (31.4%; main constituent: β-sitosterol—21.9%). Notable amounts of α-tocopherol and α-amyrin were also observed. Campesterol, 2,3-butanediol, and 3-hexanol were detected only in **SH12**. Noteworthily, the ratio of fatty acids (LA:OA:PA) in **SH1** and **SH10-SH12** remained similar (approximately 2.5:1.1:1), with a prevailing share of linoleic acid. The greatest similarities in the LA:OA:PA ratio were observed between **SH1** and **SH10** and between **SH11** and **SH12**. All compounds identified in **SH1**, **SH9**-**SH12** are listed in Table 1.

### 2.2. LC-PDA-MS Characterization of SH1-SH8

#### 2.2.1. Qualitative Analysis

Qualitative evaluation of the extracts and fractions confirmed free phenolic acids in the composition (7), (2, 4–5, 10, 13–14, 16, 18, and 20), and *p*-coumaric acid (3, 12). The flavonoids were represented in free (22, 24) and bound form (15, 19). All 24 compounds listed in Table 2 were present in the extracts and fractions, displaying selectivity to the corresponding solvent, as indicated in Figures S1 and S2 (Supplementary Materials).

#### 2.2.2. Quantitative Analysis

The quantitative assessment of apigenin and caffeoylquinic derivatives in **SH1**-**SH8** is presented in Table 3.

**Figure 1:**
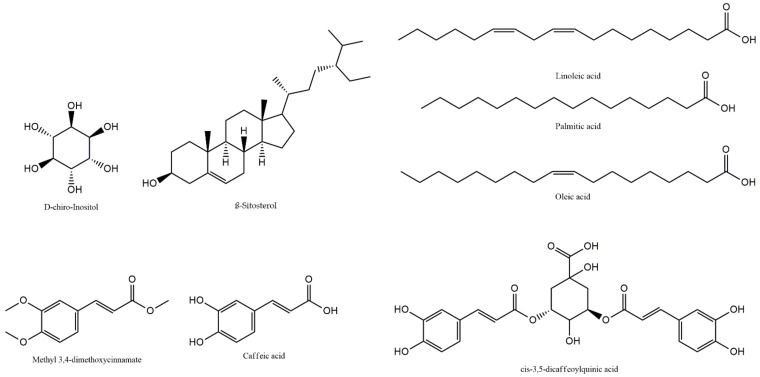
Structures of major compounds (D-chiro-inositol, β-sitosterol, methyl 3,4-dimethoxycinnamate, caffeic acid, cis-3,5-dicaffeoylquinic acid, linoleic acid, palmitic acid, oleic acid) identified in **SH1**-**SH12**.

The chemical structures of the main components of **SH1**-**SH12** detected and identified in GC-MS and LC-PDA-MS analyses are presented in Figure 1.

### 2.3. Cell Viability Assay

A preliminary cell viability test indicated that three (**SH1**, **SH4**, and **SH11**) out of the eleven obtained extracts and fractions from *S. hispanica* seeds displayed cytotoxicity against MCF-7 and MDA-MB-231 human mammary carcinoma cell lines. The remaining extracts and fractions (**SH2**-**3**, **SH5**-**SH10**) and **SH12** did not exhibit any cytotoxicity toward either breast cancer cell lines or normal skin fibroblast cells at concentrations up to 1000 µg/mL.

**Figure 2:**
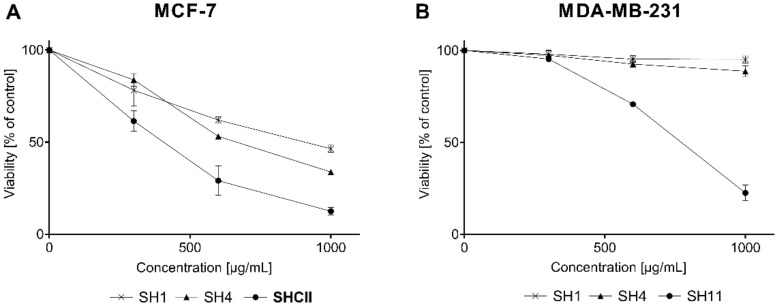
The influence of **SH1**, **SH4**, and **SH11** on the viability of MCF-7 (**A**) and MDA-MB-231 (**B**) cell lines after 24 h of incubation with increasing concentrations of the given extract and fractions (300–1000 μg/mL). Values are presented as mean ± SD from three independent experiments performed in duplicate.

Figure 2A presents the cytotoxic activity of **SH1**, **SH4,** and **SH11** against **MCF-7** cells. Figure 2B portrays the cytotoxicity of the extracts in MDA-MB-231 cells. The greatest cytotoxic activity was observed for **SH11**. IC_50_ values for the tested cell lines were 399.18 ± 54.15 μg/mL for MCF-7 and 781.26 ± 21.43 μg/mL for MDA-MB-231. **SH1** and **SH4** was active only in MCF-7 cells with IC_50_ values of 847.72 ± 69.25 μg/mL, 626.01 ± 5.07 μg/mL, respectively. Data obtained from the phytochemical analysis indicate that **SH1**, **SH4,** and **SH11** were characterized by the greatest content of potentially bioactive compounds and therefore their influence on the process of cell proliferation was evaluated. In a previous study by the research team, cisPt—a reference compound in this study—inhibited the growth of 50% of breast cancer cells at concentrations of 93 ± 2 μM for MCF-7 and 82 ± 2 μM for MDA-MB-231 [33].

### 2.4. DNA Biosynthesis Assay

**Figure 3:**
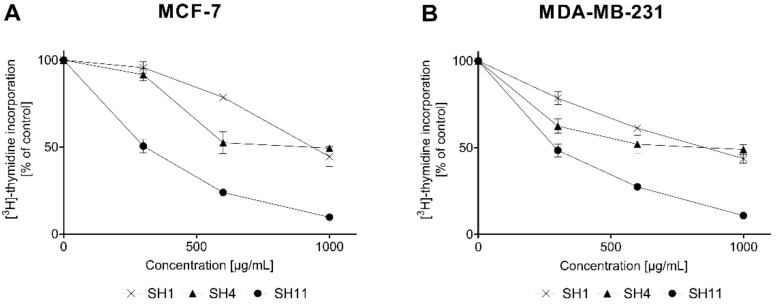
The effect of **SH1**, **SH4**, and **SH11** on the process of DNA biosynthesis in MCF-7 (**A**) and MDA-MB-231 (**B**) cell lines after 24 h of incubation with increasing concentrations of the given extract and fractions (300–1000 μg/mL). Values are presented as mean ± SD from three independent experiments performed in duplicate.

To confirm the results obtained in the preliminary cytotoxicity assay, the effect of **SH1**, **SH4**, and **SH11** on [^3^H]-thymidine incorporation in breast cancer cells was evaluated. The results are presented in Figure 3.

The results obtained in the DNA biosynthesis assay indicate that **SH11** was similarly effective as a proliferation inhibitor in both cell lines, with IC_50_ of 293.64 ± 16.61 μg/mL (MCF-7) and 265.05 ± 25.44 μg/mL (MDA-MB-231). The reference compound cisPt was previously reported to reduce the incorporation of [^3^H]-thymidine by 50% at 98 ± 2 and 86 ± 2 μM for MCF-7 and MDA-MB-231 cells, respectively [33]. Table 4 summarizes all IC_50_ values obtained in the assay.

### 2.5. Annexin V/PI Binding Assay

To examine whether the molecular mechanism of cytotoxicity of **SH1**, **SH4,** and **SH11** in MCF-7 cells was associated with their ability to induce apoptosis, an analysis of Annexin V/PI binding was performed.

**Figure 4:**
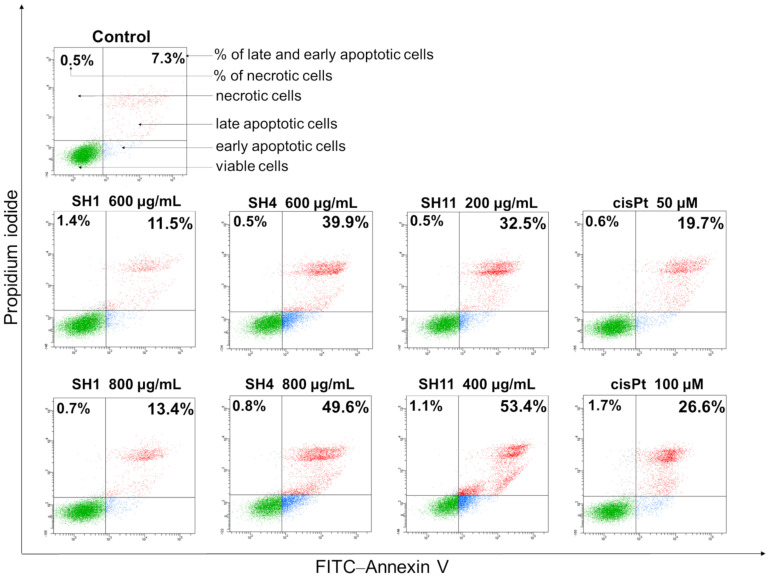
Apoptosis induction in MCF-7 breast cancer cells after 24-h incubation with **SH1**, **SH4**, **SH11**, and cisPt as a reference. The tested concentrations were 600 and 800 μg/mL for **SH1** and **SH4**, 200 and 400 μg/mL for **SH11**, and 50 and 100 μM for cisPt. The number of total early and late apoptotic cells, in addition to the number of necrotic cells, are the mean percentage from three experiments performed in duplicate.

All extracts, in addition to cisPt used as a reference, were applied at concentrations that are approximately IC_25_ and IC_50_ values evaluated in the preliminary cytotoxicity assays. The results obtained in the performed assay reveal that **SH1**, **SH4,** and **SH11** induce the apoptosis process in MCF-7 cells in a concentration-dependent manner. Figure 4 indicates that the greatest pro-apoptotic activity was exhibited by **SH11**; 24 h incubation with the extract at a concentration of 200 μg/mL resulted in the detection of 32.5% of early and late apoptotic cells. At a higher concentration (400 μg/mL), the total percentage of apoptotic cells increased to 53.4%. For **SH4**, 39.9% of apoptotic cells were detected after incubation with 600 μg/mL, and 49.6% for 800 μg/mL. **SH1** did not exhibit a similarly strong pro-apoptotic effect. Incubation for 24 h with the extract resulted in the detection of 11.5% and 13.4% of apoptotic cells for concentrations of 600 and 800 μg/mL, respectively. For cisPt, 19.7% of total apoptotic cells after incubation with 50 μM and 26.6% of early and late apoptotic cells for a 100 μM concentration of the compound were detected. The number of necrotic cells did not exceed 2% in the analyzed samples, which suggests that apoptosis, not necrosis, is the dominant mechanism of cytotoxicity for **SH1**, **SH4**, and **SH11**.

### 2.6. Western Blot Evaluation of the Expression of Apoptosis- and Autophagy-Related Protein

A deeper investigation of the pro-apoptotic and pro-autophagic effects of **SH1**, **SH4**, and **SH11** in MCF-7 cells was performed. To assess how the extract and fractions affect the expression of proteins involved in the processes of apoptosis and autophagy (BCL-2, Bax, ATG5, and LC3B), in addition to phosphorylated focal adhesion kinase (p-FAK), the Western blot technique was used.

**Figure 5:**
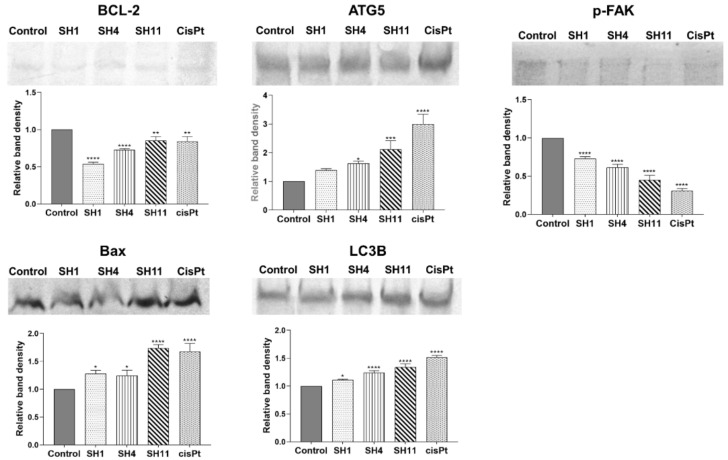
Western blot analyses of BCL-2, Bax, ATG5, LC3B, and p-FAK expression in MCF-7 cells after 24-h incubation with **SH1**, **SH4**, **SH11**, and cisPt. The tested concentrations were 800 μg/mL for **SH1** and **SH4**, 400 μg/mL for **SH11**, and 100 μM for cisPt. Results are presented as mean optical density ± SD from three measurements. Statistical significance was calculated using one-way ANOVA with Bonferroni multiple comparison test. Differences were considered statistically significant at * (*p* ≤ 0.05), ** (*p* ≤ 0.005), *** (*p* ≤ 0.0005), and **** (*p* ≤ 0.0001).

In the Western blot analyses, extracts and cisPt were applied to MCF-7 cells at concentrations that corresponded to the approximate IC_50_ values determined in the viability and DNA biosynthesis assays—for **SH1** and **SH4**: 800 μg/mL; **SH11**: 400 μg/mL. CisPt used as reference was applied at 100 μM. Results of the Western blot analyses are presented in Figure 5. **SH1**, **SH4**, and **SH11**, in addition to cisPt, were observed to exhibit the ability to inhibit the expression of pro-survival protein BCL-2 and increased the expression of apoptosis-accelerating protein Bax. For BCL-2, the greatest inhibition was observed for **SH1**. After 24 h of incubation with **SH4** and **SH11**, the attenuative activity on BCL-2 expression was observed as well. CisPt decreased the concentration of BCL-2 to a degree comparable to **SH11**. For the apoptosis regulator Bax, enhanced expression was observed in all the examined samples. The most potent activity was observed for **SH11**—the intensity of the band increased the most compared with the untreated control cells. The reference drug cisPt caused a similar effect on Bax expression. **SH1** and **SH4** enhanced Bax expression; **SH1**, **SH4**, **SH11,** and cisPt all increased the concentration of autophagy-related proteins ATG5 and LC3B. Enhancement of ATG5 expression was significant. The intensity of the **SH11** band was doubled in comparison with the control band. **SH1** and **SH4** increased the expression of ATG5 to a notable degree as well. For cisPt, the greatest enhancement in band density was observed. The expression of autophagy marker LC3B was intensified in all the analyzed samples. **SH1**, **SH4,** and **SH11** all increased the expression of LC3B compared with the untreated control cells. The reference drug cisPt increased the expression of the protein to the greatest degree, by approximately 50%. Significant inhibition of p-FAK expression was observed in all assessed extracts, in addition to cisPt. The most significant inhibitory effect was observed for cisPt and **SH11**—the relative intensity of the bands was below 50% compared with the control band for both samples.

### 2.7. Influence of SH1, SH4, and SH11 on the Expression of Proteins Related to Cell Survival and Proliferation

**Figure 6:**
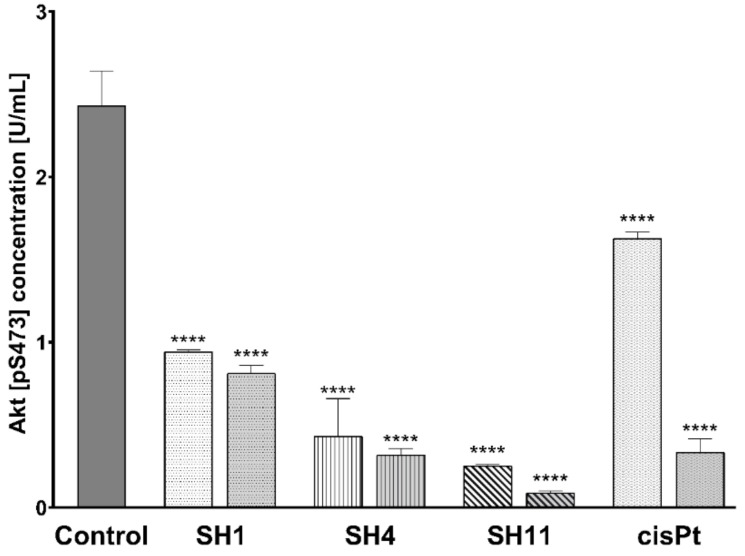
Concentrations of Akt [pS473] in MCF-7 human breast cancer cells after 24-h incubation with **SH1** and **SH4** at concentrations of 600 μg/mL and 800 μg/mL, **SH11** at 200 μg/mL and 400 μg/mL, and cisPt at 50 μM and 100 μM. Results are presented as mean ± SD from three experiments performed in duplicate. Statistical significance was calculated using one-way ANOVA with Bonferroni multiple comparison test. Differences were considered statistically significant at **** (*p* ≤ 0.0001).

An inhibitory effect on phosphorylated Akt (p-Akt) was observed in all the examined samples. As demonstrated in Figure 6, the most significant decrease was observed for **SH11**—from 2.43 U/mL in untreated control cells to 0.25 U/mL for 200 μg/mL and 0.9 U/mL for 400 μg/mL. After incubation with **SH4** at 600 and 800 μg/mL, the concentration of p-Akt was lowered to 0.43 and 0.32 U/mL, respectively. **SH1** caused a decline in p-Akt concentration to 0.94 and 0.81 U/mL, respectively. Incubation of cells with cisPt used as reference resulted in the detection of 1.63 and 0.34 U p-Akt/mL for 50 and 100 μM, respectively.

**Figure 7:**
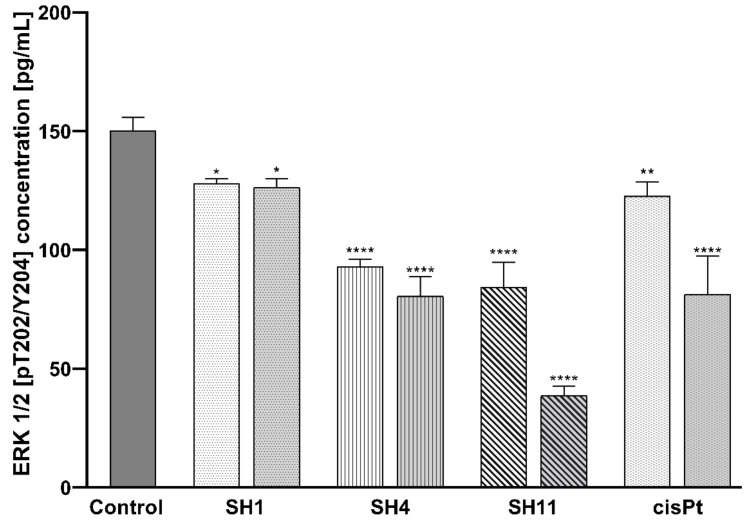
Concentrations of ERK 1/2 [pT202/Y204] in MCF-7 cells after 24-h incubation with **SH1**, **SH4**, **SH11**, and cisPt. The tested concentrations were 600 μg/mL and 800 μg/mL for **SH1** and **SH4**, 200 μg/mL and 400 μg/mL for **SH11**, and 50 μM and 100 μM for cisPt. Results are presented as mean ± SD from three independent experiments performed in duplicate. Statistical significance was calculated using one-way ANOVA with Bonferroni multiple comparison test. Differences were considered statistically significant at * (*p* ≤ 0.05), ** (*p* ≤ 0.005), and **** (*p* ≤ 0.0001).

Figure 7 presents a dose-dependent, inhibitory effect of all the analyzed *S. hispanica* extracts and fractions on the concentration of phosphorylated ERK 1/2 (p-ERK 1/2) in MCF-7 cells. In the untreated control cells, the concentration of p-ERK 1/2 was 150.33 pg/mL. The most significant decrease in p-ERK 1/2 expression was observed in **SH11**—84.33 pg/mL at 200 μg/mL and 38.67 pg/mL at 400 μg/mL. 24 h incubation with **SH4** resulted in the reduction of p-ERK 1/2 concentration to 93 pg/mL at 600 μg/mL, and 80.67 pg/mL at 800 μg/mL **SH4**. For **SH1**, the concentration of p-ERK 1/2 declined to 128 pg/mL and 126.33 pg/mL for the lower and the higher concentrations, respectively. The reference drug cisPt inhibited p-ERK 1/2 expression to 122.67 pg/mL at 50 μM and 81.33 pg/mL at 100 μM cisPt.

### 2.8. Influence of SH1, SH4, and SH11 on the Concentration of TNF-α, Interleukin-8, and Interleukin-10

The effect of **SH1**, **SH4**, and **SH11** on the concentrations of pro-inflammatory cytokines tumor necrosis factor α (TNF-α) and interleukin-8 (IL-8), in addition to anti-inflammatory interleukin-10 (IL-10), in MCF-7 cells was investigated. 

**Figure 8:**
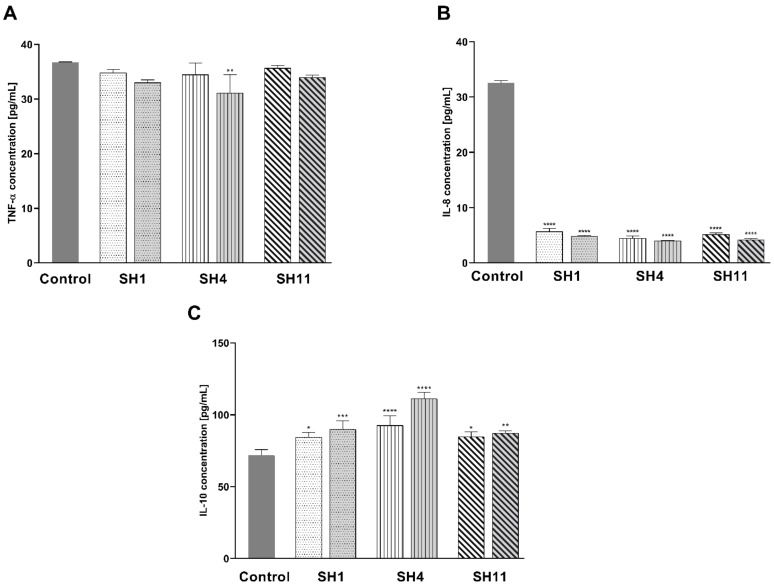
Concentrations of pro-inflammatory cytokines TNF-α (**A**) and IL-8 (**B**), and anti-inflammatory cytokine IL-10 (**C**) in MCF-7 human breast cancer cells after 24-h incubation with **SH1** and **SH4** at concentrations of 600 μg/mL and 800 μg/mL and **SH11** at concentrations of 200 μg/mL and 400 μg/mL. Results are presented as mean ± SD from three experiments performed in duplicate. Statistical significance was calculated using one-way ANOVA with Bonferroni multiple comparison test. Differences were considered statistically significant at **** (*p* ≤ 0.0001), *** (*p* ≤ 0.0005), ** (*p* ≤ 0.005), and * (*p* ≤ 0.05).

As demonstrated in Figure 8A, the inhibitory activity towards TNF-α was observed for all three extracts. For **SH1**, the concentration of this cytokine was reduced from 36.72 pg/mL in the control cells to 34.82 pg/mL and 32.99 pg/mL at the concentrations of 600 μg/mL and 800 μg/mL, respectively. At 600 and 800 μg/mL concentrations of **SH4**, the TNF-α concentration decreased to 34.46 and 31.10 pg/mL, respectively. For **SH11**, the observed concentrations of TNF-α were 35.66 pg/mL for 200 μg/mL and 33.93 pg/mL for 400 μg/mL **SH4**. As it is portrayed in Figure 8B, all three extracts caused a notable inhibition of IL-8 concentration. The inhibitory activity of **SH1**, **SH4**, and **SH11** was more significant in comparison with the control than for TNF-α. The 24-h incubation with **SH1** at 600 and 800 μg/mL resulted in a decrease in IL-8 concentration from 32.58 pg/mL in the control cells to 5.69 and 4.87 pg/mL, respectively. The analysis indicated that **SH4** decreased the concentration of IL-8 to a more notable degree with 4.48 pg/mL for 600 μg/mL and 4.00 pg/mL for 800 μg/mL. Incubation with **SH11** led to a decline in IL-8 concentration to 5.16 pg/mL for 200 μg/mL and 4.21 pg/mL for 400 μg/mL. Figure 8C illustrates that the enhancement of IL-10 concentration was observed for **SH1**, **SH4**, and **SH11**. In comparison with control cells (71.84 pg/mL), the greatest increase in the concentration of IL-10 was detected for **SH4** (92.55 pg/mL and 111.10 pg/mL for 600 and 800 μg/mL, respectively). **SH1** and **SH11** caused a similar enhancement of IL-10 concentration. 24 h incubation with 600 μg/mL **SH1** resulted in the detection of IL-10 in the concentration of 84.31 pg/mL For 800 μg/mL, the concentration of IL-10 was 89.93 pg/mL. For **SH11** at 200 and 400 μg/mL, the concentration of the cytokine went up to 84.63 and 87.26 pg/mL, respectively.

## 3. Discussion

Plant-based medicinal products can have varied effects on cancer patients, including influence on the activity of hormones and enzymes, stimulation of immune cells, or alleviating the side effects of treatment [34]. In Europe, breast cancer patients are frequent users of phytotherapy as complementary medicine along with their standard therapy [35,36]. Plant products are reported to be applied to bring physical and emotional comfort, relieve the side effects of therapy, avert tumor relapse, and improve the patient’s immune system [37]. Anti-breast cancer activity of various medicinal plants, in addition to phytochemicals isolated from them, was reported in multiple studies. The activity of some of them was not only proven in in vitro studies but also clinical trials regarding their anticancer properties were designed and conducted [38].

The chemical composition of the seeds of *S*. *hispanica* has not been previously reported in the literature. However, there are reports on the evaluation of the phytochemical composition of aerial and subaerial parts of the plant. While the dominant groups of compounds in the aerial part extracts are flavonoids and phenolic acids, the subaerial parts contain mostly phenolic acids, steroids, terpenoids, and fatty acids [9]. Linoleic acid (LA) was the primary fatty acid of **SH1** and **SH9**-**SH12**. The presence of LA was also reported in *S*. *hispanica* subaerial part ethyl acetate extract [18]. The presence of caffeoylquinic acid derivatives, including CA, 3-CQa, 4-CQa, 4,5-dCQa, and 3,5-dCQa was reported in the subaerial parts as well [17]. Those compounds have been reported in **SH1**-**SH8** as well, particularly in **SH4**.

A significant amount of *β*-sitosterol in the oil obtained from the *S. hispanica* seeds (**SH12**) indicated that the oil might possess health-promoting properties, as *β*-sitosterol is known to lower cholesterol levels, increase the activity of vitamin D, or even possess anti-breast cancer properties [39,40]. The notable amount of unsaturated fatty acids (44.8% of all constituents, 72.5% of all fatty acids) in **SH12** suggests that it can be utilized in the food industry. Unsaturated fatty acids must be delivered with food, as humans are not able to synthesize those compounds [41].

Although the yield of oil pressing was not as efficient as other oilseeds, such as lemon (*Citrus limon* L., Rutaceae) or pumpkin (*Cucurbita pepo* L., Cucurbitaceae) (approx. 33–37%), [42,43], **SH12** is an interesting product in other aspects, including attractive composition and lack of cytotoxicity at high concentrations. In the wild, *S. hispanica* grows in a warm steppe environment but is easy to cultivate in temperate climates. When cultivated, the plant is characterized by a high tolerance for low temperatures and requires extensive exposure to sunlight. Additionally, the oil pressure procedure is uncomplicated, and the yield might be improved by optimization of the process conditions in the future.

Out of 12 products obtained in this study, 3 were cytotoxic toward breast cancer cells—methanolic extract **SH1**, and fractions of methanolic extracts diethyl ether (**SH4**) and chloroform (**SH11**). Phytochemical analysis of **SH1** revealed that the primary constituents of the extract are carbohydrates. Although little is understood about the anticancer activity of sucrose (which was the primary carbohydrate in the extract), the pro-apoptotic activity might be a result of interactions between the remaining components, including inositol, CA, and QA. Phosphorylated inositol—inositol hexaphosphate—was cytotoxic toward mammary carcinoma cells and its synergy with doxorubicin and tamoxifen was observed [44]. The attenuative activity of CA on multi-drug resistance in cancer cells, including breast cancer cells, was reported as well. CA was observed to modify the estrogen receptors of MCF-7 cells [45,46]. This might suggest that the CA present in **SH1** sensitizes the cells to other extract components, and therefore enhances its activity in MCF-7 cells.

The major phytocomponents of **SH4** were phenolic acids, including CA, 4,5-dCQa, and a flavonoid apigenin (A). Phenolic acids have been reported in several papers as anti-breast cancer agents in vitro [47,48]. Apigenin, which was the major flavonoid in **SH4,** was reported to be selectively cytotoxic toward MCF-7 cells [49]. A combination of apigenin, CA, and 4,5-dCQa present in **SH4** might be responsible for its selective cytotoxicity in MCF-7 observed in this study.

The greatest inhibitory activity on the growth of breast cancer cells was observed for **SH11**. This fraction contained notable amounts of LA, conjugated linoleic acid (CLA), and cinnamic acid derivatives. All those compounds were previously observed to decrease the viability of breast cancer cells [50,51,52].

Based on preliminary viability tests and phytochemical composition, a series of assays was performed to investigate the molecular mechanism of the activity of those three products from *S. hispanica* seeds in breast cancer cells in vitro.

To assess the influence of **SH1**, **SH4**, and **SH11** on the cellular signaling pathways, the concentrations of proteins crucial in the pathways associated with cell survival: phosphorylated Akt and ERK 1/2, in addition to the expression of phosphorylated focal adhesion kinase (p-FAK), were evaluated in MCF-7 cells. The PI3K/Akt- and ERK 1/2-mediated pathways are essential for cell survival and proliferation. Phosphorylated Akt inhibits apoptosis by, among others, the inactivation of FOX proteins or BAD and the upregulation of NF-κB activation [53]. Inhibition of PI3K/Akt signaling via a decrease of phosphorylated Akt activates BAX and therefore promotes apoptosis in cells, including MCF-7 cells [54]. Approximately three out of ten human breast cancers are reported to be characterized by dysregulations in the ERK 1/2 cell signaling pathway [51]. Attenuation of ERK 1/2 phosphorylation leads to the initiation of apoptosis via the mitochondrial pathway [55]. FAK is a kinase whose expression promotes both PI3K/Akt and ERK 1/2 signaling pathways. Additionally, FAK is considered a crucial mediator, overexpressed in many breast cancer types. FAK promotes tumorigenesis and progression of breast cancer [56]. Disruption of Akt and ERK 1/2 phosphorylation caused by fruit-derived polyphenols was suggested to be related to apoptosis induction in breast carcinoma cells [57]. In this study, it was indicated that the phytochemicals present in *S*. *hispanica* seeds, particularly in **SH11**, inhibit the concentrations of phosphorylated Akt, FAK, and ERK 1/2 and therefore suppress their pro-survival activity in breast cancer cells. This correlated with the observations from the Annexin V binding assay, where 24-h incubation with **SH11** induced apoptosis in over 50% of cells. This may be due to the high content of LA, CLA, and dimethyl cinnamate. A *Cinnamomum cassia* (L.) J. Presl (Lauraceae) ethanolic extract, where major components were cinnamic acid and derivatives, decreased the viability, and promoted apoptosis in carcinoma cells [58]. In the literature, plant extracts rich in cinnamic acid derivatives was observed to activate apoptotic pathways in MCF-7 cells [50]. Additionally, LA was previously reported as pro-apoptotic for breast cancer cells via the ERK 1/2-mediated pathway [51]. CLA exhibits pro-apoptotic activity on MCF-7 cells via the intrinsic pathway [52]. However, the oil obtained from the seeds in this study did not exhibit any toxicity towards the cells, even at the highest concentration (1 mg/mL), although over 27% of its relative composition was LA. This might suggest that the cytotoxicity of **SH11** was caused by compounds other than LA or that the activity against cancer cells was an effect of synergy between the constituents of **SH11**.

To confirm the ability of **SH1**, **SH4**, and **SH11** to induce apoptosis on the mitochondrial pathway, their influence on the expression of proteins involved in the process of apoptosis, BCL-2 and Bax, was assessed. BCL-2 is a regulatory protein involved in the mitochondrial pathway of apoptosis, characterized by inhibitory activity on pro-apoptotic proteins—Bax and BAK [59]. Inhibition of BCL-2 expression leads to an increase in Bax and BAK concentrations, which consequently initiates apoptosis on the intrinsic pathway [60]. Previously, downregulation of BCL-2 and upregulation of Bax concentrations in MCF-7 cells were observed for extracts of *Cassia fistula* Linn. (Fabaceae) In the phytochemical analysis, the authors demonstrated that in *n*-butanol extract, the primary component was inositol [61]. In this study, the greatest inhibitory activity on BCL-2 was exhibited by **SH1**, which contains notable amounts of inositol. However, the greatest expression of Bax was observed in **SH11**, in which dimethyl cinnamate was one of the major constituents. Cinnamic acid derivatives were reported to possess cytotoxic and pro-apoptotic activity in cancer cells [62].

Along with apoptosis, the pro-autophagic activity of **SH1**, **SH4**, and **SH11** in MCF-7 cells was assessed by analyzing ATG5 and LC3B expression after exposure to the assessed products. Autophagy is a process of degradation of redundant or faulty cytoplasm components, in response to, among others, a deficiency in nutrients or chemotherapy. Autophagy can promote either cell survival or death. Autophagy and apoptosis may be induced in the cell simultaneously. In this case, the activation of both pathways leads to cell death [28]. In this study, it was demonstrated that **SH1**, **SH4**, and **SH11** affected the expression of proteins involved in autophagy—ATG5 and LC3B. Interactions between ATG5 and BCL-X_L_ in an autophagic cell indirectly promote apoptosis [63]. LC3B, involved in the formation of autophagosomes, is considered one of the most used autophagy markers [64]. Previously, the pro-autophagic activity of plant-derived products in breast cancer cells was reported [65]. Additionally, extract from the roots of *Bryonia multiflora* L. (Cucurbitaceae) enhanced the expression of LC3B in breast cancer cell lines. Interestingly, the major components of the extract were phenolic acids, including cynarine (1,5-di-caffeoylquinic acid), *p*-coumaric acid, and *trans*-ferulic acid [66]. Those and the derived compounds were present in **SH1**, **SH4**, and **SH11**, investigated in this study.

Progression of cancers, including breast cancers, involves pro-inflammatory cytokines as well [67]. Therefore, the effect of **SH1**, **SH4**, and **SH11** on the expression of IL-8 and TNF-α was assessed. IL-8 is a proinflammatory chemokine that is a significant factor in signaling pathways, including the ones involved in angiogenesis, proliferation, and metastasis in tumors. Inhibitory activity on IL-8 signaling is a desired effect of therapeutic agents in cancer treatment [29]. In the literature, inhibition of IL-8 concentration in breast cancer cells after exposure to plant-derived products was reported in several papers [68,69]. In the present study, all three examined *S*. *hispanica* seed extracts and fractions lowered the concentration of IL-8 in MCF-7 cell lysates to a significant degree (by approximately 90%). No previous studies on the anti-inflammatory activities of *S*. *hispanica* have been reported in the literature.

Independently, the influence of **SH1**, **SH4**, and **SH11** on another pro-inflammatory cytokine, TNF-α, was assessed. Present in the microenvironment of the tumor, it takes part in the development and metastasis of breast cancer, in addition to its relapse. TNF-α plays a dual role in breast cancer—it can promote apoptosis and proliferation in different breast cancer cell lines, however, the original cellular response to TNF-α is increased proliferation and induction of breast cancer metastasis. Therefore, TNF-α antagonists are suspected to suppress metastasis based on the results of preclinical research [30]. The in vitro investigation of the anti-inflammatory properties of several *Scorzonera* species present in Turkey revealed that aqueous methanolic extracts from the aerial parts of the plants can inhibit TNF-α production in LPS-treated leukemia cells [70]. Costantini and colleagues [71] reported that a hydrophilic fraction of the oil from pomegranate [(*Punica granatum* L. (Lythraceae)] seeds caused a decrease in viability and TNF-α concentration in breast cancer cell lines, but no significant impact on apoptosis was discovered. This study demonstrated that the extract and fractions from *S*. *hispanica* seeds inhibit TNF-α production in the cells, but only **SH4** at the higher concentration exhibits a statistically significant inhibitory activity. Contrary to the study, all the assessed products (**SH1**, **SH4**, **SH11**) exhibited pro-apoptotic properties.

*Scorzonera hispanica* seeds yielded biologically active products, particularly **SH11**. According to the chemical characterization and biological studies performed for the purpose of this study, the seeds from *S. hispanica* can be a material of wide interest, with potential applicability in the field the breast cancer treatment.

## 4. Materials and Methods

### 4.1. Chemicals and Equipment

Hexane, BSTFA:TMCS (99:1) (N,O-Bis(trimethylsilyl)trifluoroacetamide with 1% trimethylsilyl chloride), a C7-C40 *n*-alkanes calibration standard, DMSO (dimethyl sulfoxide), MTT (3-(4,5-dimethylthiazole-2-yl)-2,5-diphenyltetrazolium bromide)), TRIS (2-amino-2-(hydroxymethyl)-1,3-propanediol), and SDS (sodium dodecyl sulfate) were purchased from Sigma-Aldrich (St Louis, MO, USA). For LC-MS analysis, acetonitrile Optima (ACN) (Fisher Chemical, Loughborough, UK) and ultrapure water, freshly prepared using the system POLWATER DL3-100 system (Kraków, Poland), were used. The phase modifier formic acid (FA) was ordered from Merck. The standards apigenin (A) and 3-caffeoylquinic acid (3-CQa), 4-caffeoylquinic acid (4-CQa), 5-caffeoylquinic acid (5-CQa), 3,5-di-caffeoylquinic acid (3,5-dCQa), and 4,5-di-caffeoylquinic acid (4,5-dCQa) used for the LC-MS analysis were purchased from BIOKOM (Janki, Poland). Caffeic acid (CA) was purchased from Carl Roth (Karlsruhe, Germany), while apigenin 7-*O*-glucuronide and luteolin (purity > 98%) were previously isolated in the Department of Pharmacognosy of the Medical University of Bialystok, Poland [72,73]. Extraction of the analyzed plant material was assisted by ultrasound generated by an ultrasonic bath (Sonic-5, Polsonic, Warsaw, Poland). Extracts and fractions were filtered and concentrated to dryness under vacuum (BÜCHI system (Flawil, Switzerland)) at a controlled temperature (40 ± 2°C) and subjected to lyophilization using Lymph-Lock 1.0 (LABCONCO, Kansas City, MO, USA) vacuum concentrator until a constant weight was obtained. The seed oil was pressed using a Wartmann oil press (Ronic, Lodz, Poland). All samples were centrifuged in an MPW-380R centrifuge (MPW Med Instruments, Warsaw, Poland). Analysis of the chemical composition of the samples was performed using an Agilent Infinity 1260 liquid chromatography system coupled with a 6230 MS/TOF mass spectrometer (Agilent, Santa Clara, CA, USA). Separation was performed on Kinetex XB-C18 column (150 × 2.1 mm, 1.7 µm) (Phenomenex, Torrance, CA, USA). The 7890A GC System coupled with a Q mass spectrometer (5975C VL MSD) (Agilent Technologies, Palo Alto, CA, USA) was used for the GC-MS analysis of samples. Cell lines (MCF-7, MDA-MB-231, and human skin fibroblasts) were purchased from American Type Culture Collection (ATCC, Manassas, VA, USA). Dulbecco’s modification of eagle medium (DMEM), 1% streptomycin/penicillin mixture, phosphate-buffered saline (PBS) without calcium and magnesium, and 0.05% trypsin with 0.02% EDTA were purchased from Corning (Kennebunk, ME, USA), 10% FBS (fetal bovine serum) was purchased from Eurx (Gdansk, Poland). Hydrogen chloride (HCl) and sodium chloride (NaCl) were purchased from POCH (Gliwice, Poland). Sodium hydroxide (NaOH) and trichloroacetic acid (TCA) were purchased from Stanlab (Lublin, Poland). [^3^H]-thymidine (7 Ci/mmol) was purchased from Moravek Biochemicals (Brea, CA, USA). Tween 20 and non-fat dairy milk were purchased from BIO-RAD (Warsaw, Poland). Primary and secondary antibodies for Western blot analyses were purchased from Cell Signaling Technology (Davers, MA, USA). Round 100 mm plates and 6-well plates for adherent cell culture were purchased from Sarstedt (Nümbrecht, Germany). UV-VIS Helios Gamma Spectrophotometer (Unicam/ThermoFisher Scientific Inc., Waltham, MA, USA) was used to measure the absorbance in the cell viability assay. Radioactivity in the DNA biosynthesis assay was measured in TRI-CARB 1900TR Liquid Scintillation Counter (Packard, Perkin Elmer, Inc., San Jose, CA, USA). BD Annexin V: FITC Apoptosis Detection Kit II, (ThermoFisher Scientific Inc., Waltham, MA, USA). The analysis of Annexin V: FITC was performed with a BD FACSCanto II flow cytometer (BD Biosciences Systems, San Jose, CA, USA) using FACSDiva software (version 6.1.3, BD Biosciences Systems, San Jose, CA, USA). LKB 2117 Multiphor II Electrophoresis Unit (LKB, Stockholm, Sweden) was used to perform electrophoresis. Images of the nitrocellulose membranes were captured using Bioanalytical Imaging System Azure 280 (Azure Biosystems Inc., Dublin, CA, USA). Analysis of the images was performed with ImageJ (version 1.53, National Institute of Health, Bethesda, MD, USA). High sensitivity ELISA kit for the analysis of Akt [pS473] concentration was purchased from Invitrogen (ThermoFisher Scientific, Waltham, MA, USA). ELISA kits for the quantification of ERK 1/2 [pT202/Y204], IL-8, IL-10, and TNF-α, in addition to Sigmafast NBT/BCIP solution, were purchased from Abcam (Cambridge, UK).

### 4.2. Plant Material, Preparation of Extracts, Fractions, and Oil

*S. hispanica* seeds were purchased from W. Legutko (batch number 68347; Jutrosin, Poland). For the preparation of the extracts and fractions, *S. hispanica* seeds were broken into pieces using an electric mill. Powdered seeds (15 g, each) were then treated with ultrasound-enhanced extraction for 5 × 15 min. at 40 °C using 100 mL of solvent for each time. Finally, elimination of the solvent yielded the extracts: **SH1** (methanol) (9.1%), **SH2** (50% methanol) (10.5%), **SH3** (water) (21.3%), and **SH8** (70% acetone (*v*/*v*)) (27.9%). In addition, the fractured seeds (90 g) were continuously extracted with petrol (**SH9**; 3 L × 25 h) (15.1%), then chloroform (**SH10**; 3.5 L × 25 h) (3.2%) using the Soxhlet apparatus. Then, the cleaned source was etched with methanol (1.5 L × 26) and 50% methanol (*v*/*v*, 1.5 L × 5) for 1 h each time. The combined alcoholic extracts were suspended in water and subjected to fractioning with solvents of increasing polarity: chloroform (**SH11**; 40 × 150 mL) (0.43%), diethyl ether (**SH4**; 59 × 150 mL) (0.37%), ethyl acetate (**SH5**; 60 × 150 mL) (0.63%), and *n*-butanol (**SH6**; 34 × 150 mL) (1.28%). Water residue was filtered and treated as an additional fraction named **SH7** (1.58%). Fractions **SH1**-**SH8** were freeze-dried. Cold pressing the seeds (150 g, triplicate, at 35 °C) provided the oil (**SH12**). The crude oil was then centrifuged (2000 rpm, 10 min, at 25 °C) and then separated from the precipitate. The pressing procedure yielded 4.8 mL of the oil (3.2%).

### 4.3. GC-MS Analysis of SH1, SH9-SH12

For GC-MS analysis, 15 mg of **SH9** was diluted three times with hexane. However, to prepare the **SH1**, **SH10**-**SH12** samples, derivatization to trimethylsilyl (TMS) derivatives was performed. For this purpose, 200 µL of BSTFA:TMCS (99:1) was mixed with 15 mg of the dry residue of the samples. The reaction mixture was then sealed and heated at 80 °C for 30 min. The **SH9** and TMS derivatives of **SH1, SH10**, and **SH11** were analyzed on a GC System coupled with a Q mass spectrometer with a source of electron ionization (EI) (the energy of ionization was 70 eV). Chromatographic separation was performed on an HP-5ms capillary column (internal diameter: 0.25 mm, film thickness: 0.25 µm, length: 30 m, Agilent Technologies), equipped with electronic pressure control and a split/splitless injector. The helium flow rate through the column was 1 mL min^−1^ in constant flow mode. The injector (300 °C) worked in split mode (split ratio 1:10). The injection volume was 1 µL. The initial temperature of the column was 40 °C, increased by 3 °C/min until 300 °C was reached, and maintained at 300 °C for 15 min. The MSD detector acquisition parameters were as follows: transfer line temperature—300 °C; and the MS source temperature—230 °C. Detection was performed in full scan mode from 40 to 850 amu [74]. Subsequently to integration, the calculation of the fraction of separated components in the total ion current (TIC) was performed.

### 4.4. Identification of the Chemical Composition of SH1, SH9-SH12

Both mass spectral data and the calculated retention indices (RI) were utilized in the identification of the compounds. The calculation of linear-temperature-programmed RI was done from the equation:

$$RIx=100n+100\frac{t_{R\left(x\right)}-t_{R\left(n\right)}}{t_{R\left(n+1\right)}+t_{R\left(n\right)}}$$

where $t_{R\left(x\right)}$ is the retention time of the analyzed compound (*x*) and $t_{R\left(n\right)}$ and $t_{R\left(n+1\right)}$ are retention times of *n*-alkanes leaving the chromatographic column before and after the under consideration.

Therefore, the dichloromethane solution of C_7_-C_40_ *n*-alkanes was previously separated under the above-mentioned conditions. The MS libraries used were Wiley and NIST [75]. The MS library was searched using a probability-based matching algorithm. Other literature was used to identify individuals [76,77,78,79,80]. The percentage of individual component relative number was presented as percent peak area relative to total peak area (%) (semiquantitative analysis).

### 4.5. LC-PDA-MS Conditions

Separation and qualitative evaluation of the extracts were performed on a C18 column using a liquid chromatograph. The qualitative and quantitative assessments were done under the following conditions: eluent A and B: UPW and ACN with 0.1% FA, respectively, flow rate: 300 μL/min; thermostat temperature 25 ± 0.8 °C; chromatogram wavelength 325 and 340 nm, UV-Vis spectrum at range 190–500 nm, injection: 1 μL. The gradient starts from 5 min of the 5% B starting condition and forms two isocrats—18% B between 15–40 min and 65% between 72–80 min with corresponding increments. Equilibration was 10 min. The MS/TOF conditions were as follows: the flow of dying and shielding—12 L/min at 350 °C. The nebulizer pressure was set at 45 psi, the capillary voltage at 2500 V with nozzle voltage 1000 V for negative ion mode. The acquisition was set at 120–1700 m/z controlled by Mass Hunter Data Acquisition 10.1. Data analysis was performed in Mass Hunter Qualitative b10.0 with a ChemStation integrator.

### 4.6. LC-PDA-MS Optimization and Validation

#### 4.6.1. Preparation of Standard Solutions and Samples

The 5CQa and A were prepared in 50% MeOH, then filtered through a 0.45-μm PVDF membrane. Final solutions were achieved through the serial dilution of stock solution in volumetric flasks with the initial phase position. The working concentration range was 0.5–100 µg/mL and 2.5–100 µg/mL for 5CQa and A, respectively. Samples were prepared by carefully making aliquots, dissolving, centrifuging, and diluting in the initial mixture of phases to 1 mg/mL.

#### 4.6.2. Chromatographic Optimization

Separation optimization allowed for the separation of substances confirmed using PDA and MS detectors. The linearity of the detector operation was assessed with a satisfactory result. Limits of detection (LOD) and quantification (LOQ) were plotted from the value of dividing the standard error of the response by slope. For LOD, multiplication by 3.3 was assumed, and for LOQ, 10 times this value. The validation process meets the ICH standards [81] and the parameters are summarized in Table 5.

### 4.7. Cell Culture

The cell culture medium was DMEM (10% FBS and a 1% streptomycin/penicillin mixture were added to the medium). Cell culture was maintained in 100 mm plates and placed in an incubator in the proper conditions: 37 °C, 5% CO_2_, and 90% humidity. After the achievement of desired confluence (approximately 85%), the cells were detached from the plate using PBS and 0.05% trypsin with 0.02% EDTA. Then, the cells, suspended in DMEM, were transferred to six-well plates with a density of 5 × 10^5^ cells per well. After 24 h of incubation in six-well plates, the cells were used for the assays presented below.

### 4.8. Cell Viability Assay

The investigate how **SH1**-**SH12** affect the viability of selected cell lines, the MTT assay was performed following the modified method introduced by Carmichael et al. [82]. Cells were seeded and cultured in 6-well plates, as described in Section 4.7. Then, the cells were incubated with increasing concentrations of **SH1**-**SH12** (up to 1000 μg/mL) in duplicate. After incubation with MTT, the solution was aspirated and DMSO was added to dissolve formazan crystals. The absorbance (read at 570 nm) in each well was referred to the untreated control cells (taken as 100%) and expressed as a percent of the mean control value, according to the method described in the previous study by the research team [83].

### 4.9. DNA Biosynthesis Assay

The extract and fractions selected in the preliminary cytotoxicity assay (**SH1**, **SH4**, **SH11**) were assessed in the DNA biosynthesis assay where the amount of [^3^H]-thymidine incorporated into the DNA of cells is measured as described in the previous study [84]. The mean radioactivity of untreated control wells was considered 100%. Values observed in the tested wells were expressed as a percent of the mean control value.

### 4.10. Flow Cytometry Evaluation of Annexin V Binding

To assess the ability of **SH1**, **SH4**, **SH11**, and cisplatin (cisPt) to induce apoptosis in MCF-7 cells, a flow cytometric assay with Annexin V-FITC Apoptosis Detection Kit II was performed, according to the producer’s protocol. In brief, after 24 h of incubation with various concentrations of the tested extracts and reference drug, the cells were transferred from 6-well plates to test tubes and suspended in a binding buffer. Annexin V-FITC and PI (propidium iodide) (5 μL, each) were added to each sample and the mixtures were subsequently incubated at room temperature for 15 min. The analysis was performed using a flow cytometer and FACSDiva software.

### 4.11. Analysis of Protein Expression Using Western Blot Technique

Cell lysate samples (**SH1** and **SH4**: 800 μg/mL, **SH11** 400 μg/mL, cisPt 100 μM) containing 30 μg of protein each were subjected to SDS-PAGE. The electrophoresis was run at 100 V for 1.5 h.

The protein transfer to nitrocellulose membranes was done in the electrophoresis unit (1 h at 20 mA). After the transfer, nitrocellulose was washed with 5% non-fat dairy milk in TBS-T (TRIS-buffered saline with Tween 20 (20 mM TRIS-HCl buffer, pH 7.6, with 150 mM NaCl and 0.05% Tween 20)) for 1 h. Subsequently, overnight incubation of membranes with monoclonal antibodies against BCL-2, Bax, ATG5, LC3B, and p-FAK in TBS-T took place. Then, secondary alkaline phosphatase-conjugated antibodies against rabbit immunoglobulin (1:1000) diluted in TBS-T were added to each nitrocellulose membrane and 1 h of incubation with gentle shaking took place. After the incubation, the nitrocellulose membranes were washed with TBS-T four times and exposed to Sigmafast BCIP/NBT in the darkness. Images of the nitrocellulose membranes were subsequently captured and analyzed.

### 4.12. Analysis of Protein Concentration Using ELISA Technique

The evaluation of the protein concentrations (p-Akt, p-ERK 1/2, IL-8, IL-10, TNF-α) in MCF-7 cell lysates was done using high-sensitivity assay kits. Cell lysates were obtained and stored as described previously [85]. Untreated cells acted as the control. All tests were performed according to the producer’s protocols, on microplates precoated with specific antibodies, provided with the kits.

### 4.13. Statistical Analysis

Data from three replicates are summarized as mean ± standard deviation (SD). Statistical analysis was done in GraphPad Prism Version 6.0 (GraphPad Software, Inc., San Diego, CA, USA). The one-way ANOVA with Bonferroni multiple comparison test was performed to calculate the differences between the results obtained in the control and tested cells, in addition to linear regression parameters confirming their statistical significance. Calculations for regression parameters were made using MS Excel 2019. Statistically significant differences were defined as *p <* 0.05.

## 5. Conclusions

The results obtained throughout this study demonstrate that *S. hispanica* seeds, particularly the oil, are a source of multiple natural products, including saturated and unsaturated fatty acids, and phytosterols. **SH12** might be a product of special interest in the future. The procedure of oil cold pressing is uncomplicated and this product exhibits no cytotoxicity toward cells in vitro. However, extracts and fractions obtained from *S. hispanica* seeds contain multiple bioactive compounds such as polyphenols including quinic and cinnamic acid derivatives, and apigenin, but also fatty acids and organic polyols. In the biological assays, **SH1**, **SH4,** and **SH11** exhibited cytotoxic activity in the MCF-7 human mammary carcinoma cell line via the inhibition of the PI3K/Akt and ERK 1/2 cell signaling pathways. **SH1**, **SH4**, and **SH11** were also observed to alter the expression of proteins related to both apoptosis and autophagy. Their inhibitory activity on IL-8 expression may lead to the suppression of angiogenesis and tumor metastasis. Nevertheless, an in-depth investigation of the activity of the extracts, in addition to their constituents, is required. So far, the results obtained in this study might suggest that *S*. *hispanica* seeds are a promising source of bioactive compounds that could potentially find use in breast cancer therapy.

## Figures and Tables

**Table 1:** GC-MS analysis of compound groups identified in **SH1**, **SH9**-**SH12**.

Compounds	Analytical Parameters				Relative Composition, %				
	RI^Exp^	RI^Lit^	Target Ions, m/z	M^+^	SH1	SH9	SH10	SH11	SH12
Organic Acids					11.2	41.7	53.0	41.0	62.2
***Fatty acids***					**7.5**	**41.7**	**44.2**	**33.0**	**61.8**
Linoleic acid (LA, 18:2), mono-TMS	2220	2215	73 (100), 75 (99), 337 (86), 67 (62), 81 (59)	352	3.7	38.7	18.8	9.9	27.2
Oleic acid (OA, 18:1), mono-TMS	2225	2220	339 (100), 73 (93), 117 (92), 75 (86), 129 (76)	354	1.4		9.0	6.5	16.0
Palmitic acid (PA, 16:0), mono-TMS	2054	2052	313 (100), 117 (91), 73 (71), 75 (51), 132 (42)	328	1.2		6.9	5.9	15.7
Conjugated linoleic acid (CLA, 18:2), mono-TMS			73 (100), 75 (83), 117 (62), 129 (50), 105 (48)	352	0.3	2.1	0.4	2.4	
***Aliphatic monocarboxylic acids***					**0.1**		**1.4**	**1.6**	**0.4**
Hexanoic acid (6:0), mono-TMS	1078	1071	75 (100), 173 (87), 73 (85), 117 (38), 131 (15)	188	0.02		0.8	0.8	0.4
Nonanoic acid (9:0), mono-TMS	1366	1358	73 (100), 75 (76), 215 (74), 117 (61), 129 (25)	230			0.2	0.4	
Octanoic acid (8:0), mono-TMS	1269	1262	73 (100), 75 (86), 201 (84), 117 (60), 129 (23)	216	0.03		0.2	0.2	
***Aliphatic dicarboxylic acid***					**1.2**		**6.1**	**2.2**	
Glutaric acid, di-TMS	1388	1394	73 (100), 147 (85), 131 (41), 103 (23), 59 (16)	276	0.3		4.1	0.4	
Azelaic acid, di-TMS	1810	1812	73 (100), 75 (78), 317 (53), 201 (41), 129 (33)	332			1.0	0.7	
Malic acid, tri-TMS	1513	1510	73 (100), 147 (70), 233 (26), 245 (16), 133 (12)	350	0.6				
Sebacic acid, di-TMS	1907	1905	73 (100), 75 (76), 331 (65), 215 (36), 129 (34)				0.3	0.4	
***Aromatic acids***					**0.03**		**0.3**	**0.7**	
Benzoic acid, mono-TMS	1247	1248	179 (100), 147 (81), 105 (69), 135 (48), 77 (46)	194	0.03		0.1	0.2	
Phenylacetic acid, mono-TMS	1300	1302	73 (100), 75 (32), 164 (19), 91 (17), 193 (16)	208				0.2	
Salicylic acid, di-TMS	1518	1513	73 (100), 267 (46), 147 (29), 103 (24), 75 (19)				0.2		
***Aliphatic hydroxy acids***					**0.6**		**0.6**	**0.6**	
L (+)-Lactic acid, di-TMS	1073	1073	73 (100), 147 (77), 117 (62), 198 (18), 191 (15)		0.1		0.2		
β-Lactic acid, di-TMS	1155	1145	147 (100), 73 (31), 177 (18), 119 (17), 148 (15)		0.03		0.2		
Glycolic acid, di-TMS	1086	1085	147 (100), 73 (76), 148 (17), 66 (16), 133 (11)		0.05			0.2	
2-Isopropyl-3-ketobutyrate, di-O-TMS	1461	1463	73 (100), 273 (80), 147 (42), 155 (31), 183 (22)	288				0.25	
Glyceric acid, tri-TMS	1351	1350	73 (100), 147 (69), 189 (39), 292 (31), 199 (27)		0.18			0.17	
***Aromatic hydroxy acids***					**1.8**		**0.2**	**2.0**	
Quinic acid, penta-TMS	1899	1902	73 (100), 345 (60), 147 (32), 75 (27), 255 (25)		1.5		0.1		
Shikimic acid, tetra-TMS	1844	1845	252 (100), 73 (88), 204 (83), 131 (56), 103 (36)					1.2	
Hydroxybenzoic acid, di-TMS	1623	1623	73 (100), 267 (42), 193 (33), 282 (32), 103 (13)	282	0.02			0.5	
***α*,*β-Unsaturated carboxylic acids***					**0.1**		**0.1**	**0.9**	
Cinnamic acid, mono-TMS	1546	1549	205 (100), 131 (81), 103 (54), 161 (50), 73 (47)	220				0.4	
E-*p*-Coumaric acid, di-TMS	1947	1947	73 (100), 293 (81), 219 (87), 308 (69), 249 (45)	308	0.1		0.1	0.4	
**Organic esters, carbonyl compounds**					**0.5**	**20.1**	**3.8**	**27.9**	
***Fatty acid esters***					**0.3**	**18.8**	**2.7**	**4.3**	
Methyl linolelaidate	2095	2095	73 (100), 67 (56), 81 (48), 95 (33), 55 (33)	280	0.1		0.5	0.9	
9-Octadecenoic acid, 18-TMS, methyl ester	2434	2435	73 (100), 225 (55), 75 (42), 130 (27), 369 (19)	384	0.2		0.7	0.3	
Butyl 9,12-octadecadienoate	2470	2478	67 (100), 81 (84), 55 (62), 95 (58), 79 (56)	336		8.8			
Octadecadienoic acid (CLA), ester	2474	-	55 (100), 67 (76), 81 (71), 69 (60), 95 (56)	356		5.7			
***α*,*β-Unsaturated carboxylic esters***					**0.04**			**22.8**	
Cinnamic acid, 3,4-di-TMS, methyl ester	2020	2018	219 (100), 238 (58),73 (41), 220 (17), 339 (16)	338	0.04			21.16	
Cinnamic acid, methyl ester	1856	1858	252 (100), 73 (76), 179 (71), 166 (64), 209 (64)					1.19	
***Carbonyl compounds***					**0.2**	**1.3**	**1.1**	**0.8**	
2,4-Decadienal, (E, E)-	1316	1315	81 (100), 41 (16), 67 (13), 83 (11), 55 (10)	152	0.2	0.5	0.9		
Benzaldehyde, 3,5-dimethoxy-4-[(TMS)oxy]-	1708	1711	224 (100), 239 (43), 223 (29), 254 (27), 73 (25)	254				0.2	
2′,4′-Dihydroxyacetophenone, di-TMS, ether	1703	1709	73 (100), 194 (94),70 (61), 281 (53), 44 (52)	296				0.2	
2,4-Decadienal, (E, Z)-	1292	1291	81 (100), 41 (23), 83 (18), 67 (18), 55 (15)	152		0.3			
2-Decennial, E-	1260	1262	70 (100), 55 (88), 41 (82), 43 (74), 83 (68)			0.2			
Organic alcohols, diols, polyols					**16.3**	**0.8**	**19.5**	**4.1**	**0.2**
***Organic alcohols***					**0.3**	**0.8**	**0.1**	**4.1**	**0.2**
5-Allyl-1-methoxy-2,3-dihydroxybenzene, di-TMS, ether	1953	1950	73 (100), 324 (91), 293 (56), 394 (39), 204 (38)	324	0.1			3.7	
3-Heptene, 4-ol, mono-TMS	981	986	171 (100), 172 (17), 73 (10), 173 (8), 78 (7)	186	0.2				
2-Phenylethanol, mono-TMS	1229	1227	73 (100), 103 (42), 179 (40), 75 (35), 77 (21)	194			0.1		
Z,E-2,13-Octadecadien-1-ol	2071	2076	99 (100), 67 (68), 55 (66), 79 (59), 81 (58)			0.5			
3-Hexanol, mono-TMS	998	994	75 (100), 159 (95), 73 (46), 103 (21), 77 (17)						0.2
***Diols***					**0.1**		**1.6**		**0.3**
Ethylene glycol, di-TMS	998	992	147 (100), 73 (41), 191 (15), 148 (15), 103 (13)		0.05		0.9		
Propylene glycol, di-TMS	1014	1013	117 (100), 147 (66), 73 (64), 66 (11), 148 (10)		0.02		0.5		
2,3-Butanediol, di-TMS, rac	1049	1049	147 (100), 73 (97), 174 (24), 262 (18), 77 (17)						0.3
***Polyols***					**15.8**		**17.8**		
Glycerol, tri-TMS	1296	1295	147 (100), 73 (95), 205 (83), 117 (40), 103 (32)		2.1		15.7		
Inositol, Hexa-OTMS, D-chiro-	1999	1996	318 (100), 305 (85), 73 (75), 217 (71), 147 (53)	612	7.2				
D-(+)-Arabitol, Penta-TMS	1759	1760	73 (100), 217 (92), 147 (56), 103 (48), 205 (43)		2.0		0.3		
***Carbohydrates***					**54.6**		**5.4**	**0.1**	
Sucrose, octa-OTMS	2712	2712	361 (100), 73 (56), 117 (40), 362 (33), 147 (21)		18.0		2.9		
Maltose, octa-TMS, methyloxime (isomer 2)	2731	2733	73 (100), 361 (88), 217 (75), 289 (64), 147 (36)		5.4				
α-D-Fructofuranose, penta-TMS	1845	1843	73 (100), 217 (89), 147 (34), 437 (26), 218 (17)		3.8		0.3		
β-D-Fructofuranose, penta-TMS	1856	1854	217 (100), 73 (71), 437 (31), 147 (30), 218 (21)		3.0				
***Phosphorous/Organophosphorous compounds***					**1.6**		**10.8**	**3.8**	
Phosphoric acid, tri-TMS	1292	1285	299 (100), 300 (25), 73 (21), 314 (17), 301 (14)	314	0.9		8.2	3.1	
Phosphoric acid, di-TMS monomethyl ester	1192	-	241 (100), 242 (17), 133 (13), 73 (12), 211 (11)	256	0.01		1.3	0.3	
Phosphoric acid, di-TMS, 2,3-di[(TMS)oxy]propyl ester	1799	1793	73 (100), 357 (56), 299 (48), 147 (36), 129 (21)		0.8		0.3		
***Phenols***					**3.1**		**0.8**	**6.5**	
E-Ferulic acid, di-TMS	2102	2104	73 (100), 338 (89), 323 (48), 322 (42), 309 (39)	338	0.1		0.2	4.2	
Caffeic acid, tris-TMS	2158	2159	396 (100), 219 (94), 73 (65), 397 (36), 381 (24)	396	2.7				
Vanillin, mono-TMS	1538	1545	194 (100), 193 (51), 209 (47), 224 (27), 73 (24)	224			0.1	0.5	
Vanillic acid, di-TMS	1777	1776	297 (100), 267 (71), 73 (68), 312 (56), 223 (54)	312	0.04		0.1	0.3	
***Sterols***					**1.4**	**13.5**	**2.3**	**3.3**	**31.4**
β-Sitosterol, mono-TMS	3348	3342	129 (100), 357 (56),73 (58), 396 (54), 81 (47)	486	0.7	8.0	1.9	1.3	21.9
Stigmasterol, mono-TMS	3290	3286	55 (100), 83 (91), 81 (78), 73 (75), 67 (67)	484	0.2	1.1	0.5	0.7	2.6
5α-Stigmast-7-en-3β-ol	3359	3355	414 (100), 255 (85), 55 (53), 81 (50), 43 (46)	414		1.6			
5α-Stigmast-7-en-3β-ol, mono-TMS	3401	3401	73 (100), 255 (92), 487 (79), 147 (45), 229 (20)	486					4.8
Campesterol, mono-TMS	3253	3251	73 (100), 129 (62), 343 (36), 147 (30), 382 (23)	472					2.1
***Amino acids***					**3.0**		**0.6**		**0.3**
L-Proline, di- TMS	1303	1302	142 (100), 73 (28), 143 (14), 147 (7), 216 (5)	259	0.9		0.2		
Pyroglutamic acid, di-TMS	1532	1524	156 (100), 73 (60), 147 (28), 157 (13), 217 (13)	273	0.5		0.3		
Threonine, tri-TMS	1406	1408	73 (100), 218 (60), 219 (54), 117 (41), 147 (30)		0.3				
***Glycerolipids***					**0.9**	**3.3**	**1.8**	**4.5**	
2-Monolinolenin, di-TMS	2776	2780	129 (100), 73 (97), 147 (60), 103 (48), 67 (41)	498	0.5		1.1	2.1	
2-Monoolein, di-TMS	2742	2744	103 (100), 73 (84), 129 (79), 67 (43), 55 (35)		0.2		0.5	1.5	
Glycerol 1-monolinolate	2688	2697	67 (100), 81 (88), 55 (74), 95 (62), 79 (56)	354		2.8			
***Tocopherols***					**0.2**	**2.6**	**0.2**		**2.4**
(+)-α-Tocopherol, OTMS-	3152	3156	502 (100), 73 (80), 237 (68), 55 (51), 67 (41)	502	0.2		0.2		2.4
α-Tocopherol	3130	3130	165 (100), 430 (86), 164 (31), 431 (28), 166 (12)	430		2.3			
α-Tocopheryl acetate	3141	3132	165 (100), 55 (67), 430 (61), 67 (59), 81 (56)			0.3			
***Nucleosides***					**1.0**		**0.2**		
Cytidine, 2′,3′,5′-tri-TMS ether	2822	2811	73 (100), 217 (69), 147 (34), 147 (28), 151 (24)	459	0.5				
5-Methyluridine, tri-TMS derivative	2429	2428	73 (100), 217 (81), 75 (34), 55 (30), 67 (25)	474	0.2		0.2		
Uridine, 2′,3′,5′-tri-OTMS	2461	2469	73 (100), 217 (62), 103 (23), 259 (21), 147 (21)	460	0.2				
***Terpenoids***					**0.2**	**6.2**			**0.2**
***Monoterpenes, Monoterpenoids***						**0.6**			
*p*-Menthane, trans-	984	978	97 (100), 55 (67), 41 (20), 96 (20), 57 (19)	140		0.2			
Carvone	1241	1242	82 (100), 54 (38), 108 (36), 93 (36), 107 (25)	150		0.2			
Camphor	1143	1143	95 (100), 81 (86), 67 (64), 152 (59), 55 (54)	152		0.1			
***Triterpenes***					**0.2**	**5.4**			**2.0**
α-Amyrin, mono-TMS	3384	3382	218 (100), 73 (35), 189 (25), 190 (20), 219 (19)	498	0.2				2.0
α-Amyrin	3376	3376	218 (100), 207 (22), 95 (22), 135 (22), 203 (22)	426		4.2			
β-Amyrin	3330	3337	218 (100), 203 (48), 207 (26), 55 (24), 81 (23)	426		1.2			
***Sesquiterpenes*, *Sesquiterpenoids***						**0.2**			
α-Longipinene	1363	1360	41 (100), 55 (96), 43 (87), 91 (78), 44 (71)	204		0.1			
β-E-Caryophyllene	1415	1416	41 (100), 91 (99), 105 (93), 55 (91), 79 (89)	204		0.1			
**Hydrocarbons**						**4.8**			
*Aliphatic hydrocarbons*						3.9			
*Alicyclic hydrocarbons*						0.6			
*Aromatic hydrocarbons*						0.3			
**Flavonoids**								**0.4**	
**Other compounds**					**1.2**	**1.1**	**0.8**	**1.9**	**0.4**
**Non-identified compounds**					**3.5**	**6.0**	**2.3**	**6.5**	**0.6**

**Table 2:** LC-PDA-TOF/MS qualitative analysis of extracts and fractions of *S. hispanica* seeds.

No	Retention Time [min]	UV λ max [nm]	[M-H]-[m/z]	Compound Name
1	13.46	290 sh, 325	250, 300, **310**	unknown
2	18.23	290 sh, 326	**353**	5-CQa ^S^
3	19.16	290 sh, 340	**339**	3-p-CoumQa
4	21.61	250, 290 sh, 325	191, **353**, 705	3-CQa ^S^
5	22.45	290 sh, 325	**353**	4-CQa ^S^
6	23.06	295 sh, 325	**292**	unknown
7	23.27	295 sh, 326	**179**	CA ^S^
8	23.91	325	**306**	unknown
9	24.89	325	**530**	unknown
10	28.65	310	**367**	methylated 4-CQa
11	31.83	295 sh, 325	**435**	unknown
12	45.38	300 sh, 325	133, 161, 387, **549**	p-CoumQA derivatives
13	48.73	295 sh, 325	147, 353, **515**	3,5-dCQa ^S^
14	50.26	295 sh, 325	353, **515**	cis-3,5-dCQa
15	52.39	265, 338	269, **445**	apigenin 7-O-glucuronide ^S^
16	53.47	295 sh, 325	353, **515**	4,5-dCQa ^S^
17	57.16	295 sh, 325	507	unknown
18	57.47	295 sh, 320	529	methylated-diCQa
19	58.42	265 sh, 338	268, 459	luteolin 7-O-glucuronide
20	58.76	295 sh, 320	339, **529**	methylated-diCQa
21	59.26	295 sh, 328	437	unknown
22	59.58	265 sh, 338	285	luteolin ^S^
23	60.26	295 sh, 325	353, 515, **677**	triCQa
24	63.17	265, 340	151, **269**	apigenin (A) ^S^

^S^—comparisons with chemical standards were made, sh—value on the deflection of the UV spectrum, bold—most abundant ion.

**Table 3:** Assessment of apigenin and caffeoylquinic derivatives content in extracts (SH1-SH3, SH8) and fractions (SH4-SH7) of *S. hispanica* seeds.

No.	Content mg Per g of Extract/Fraction ^a^							
	SH1	SH2	SH3	SH4	SH5	SH6	SH7	SH8
2	nd	blq	nd	nd	nd	blq	blq	blq
4	3.80 ± 0.03	12.37 ± 0.18	blq	blq	nd	54.34 ± 0.13	6.23 ± 0.06	13.39 ± 0.18
5	nd	nd	nd	nd	2.90 ± 0.10	2.25 ± 0.03	blq	nd
7	nd	nd	1.16 ± 0.03	20.97 ± 0.07	nd	nd	nd	nd
10	nd	nd	nd	4.87 ± 0.07	nd	nd	nd	nd
14	7.75 ± 0.04	13.57 ± 0.19	blq	36.54 ± 0.2	242.00 ± 0.20	13.54 ± 0.13	nd	20.54 ± 0.33
15	nd	nd	nd	5.55 ± 0.7	nd	nd	nd	nd
16	3.96 ± 0.17	8.48 ± 0.09	nd	nd	nd	48.18 ± 0.41	nd	10.36 ± 0.12
17	blq	blq	nd	16.82 ± 0.49	55.6 ± 0.20	10.31 ± 0.13	nd	blq
19	nd	nd	nd	1.60 ± 0.04	nd	nd	nd	nd
21	nd	nd	nd	blq	nd	nd	nd	nd
24	blq	blq	nd	13.98 ± 0.37	6.00 ± 0.10	nd	nd	0.80 ± 0.29
25	1.98 ± 0.09	1.10 ± 0.15	blq	5.90 ± 0.11	nd	nd	nd	blq
**Total CQa**	**11.55**	**25.95**	**1.16**	**100.33**	**306.5**	**80.44**	**6.23**	**34.73**

^a^—content expressed as mean with standard deviation; blq—below the limit of quantification; nd—not detected.

**Table 4:** The influence of **SH1**, **SH4**, and **SH11** on the DNA biosynthesis in MCF-7 and MDA-MB-231 cell lines.

Sample Name	IC_50_ for MCF-7 [μg/mL]	IC_50_ for MDA-MB-231 [μg/mL]
**SH1**	943.23 ± 55.5	863.21 ± 35.81
**SH4**	630.52 ± 64.96	648.61 ± 182.62
**SH11**	293.64 ± 16.61	265.05 ± 25.44

Results are presented as mean IC_50_ values ± SD from three independent experiments performed in duplicate.

**Table 5:** Validation parameters for CQAs and A derivatives analysis by LC-MS.

Parameter	5CQa	A
Linear Range [µg/mL]	0.5–100	2.5–100
r^2^ (*n* = 6)	0.9998	0.9995
Regression Equation ^a^	*y* = 19.692*x* + 10.238	*y* = 11.494*x* + 7.493
LOD [µg/mL]	0.64	0.91
LOQ [µg/mL]	1.92	2.76
Accuracy [%]	101.45 ± 4.47	101.77 ± 6.59
Intraday precision (%CV) (*N* = 6)	1.28	0.97
Interday precision (%CV) (*N* = 9)	1.82	0.87

^a^—the value for y corresponds to the peak area and x to the concentration, respectively.

## Data Availability

The datasets used and/or analyzed during the current study are available from the corresponding author upon reasonable request.

## Supplementary Materials

The supporting information can be downloaded at: https://www.mdpi.com/article/10.3390/ijms231911584/s1

## Abbreviations

3:5-dCQa	3:5-di-caffeoylquinic acid
3-CQa	3-caffeoylquinic acid
4,5-dCQa	4,5-di-caffeoylquinic acid
4-CQa	4-caffeoylquinic acid
5-CQa	5-caffeoylquinic acid
A	Apigenin
ACN	Acetonitrile
Akt	Protein kinase B
ATG5	Autophagy-related protein 5
BAK	BCL-2 homologous antagonist
Bax	BCL-2-like protein 4
BCL-2	B-cell lymphoma 2
BCL-xL	B-cell lymphoma extra-large
CA	Caffeic acid
cisPt	Cisplatin
CLA	Conjugated linoleic acid
DMEM	Dulbecco’s modification of Eagle medium
EDTA	Ethylenediaminetetraacetic acid
EI	Electron ionization
ERK	Extracellular signal-regulated kinase
FA	Formic acid
FAK	Focal adhesion kinase
FBS	Fetal bovine serum
GC-MS	Gas chromatography-mass spectrometry
IC_25_	Quarter-maximal inhibitory concentration
IC_50_	Half-maximal inhibitory concentration
IL-8	Interleukin 8
LA	Linoleic acid
LC-PDA-MS	Liquid chromatography-photodiode array-mass spectrometry
LC3B	Light chain 3B
LOD	Limit of detection
LOQ	Limit of quantification
MTT	3-(4,5-dimethylthiazol-2-yl)-2,5-diphenyl-2H-tetrazolium bromide
NF-κB	Nuclear factor kappa B
OA	Oleic acid
PA	Palmitic acid
PAGE	Polyacrylamide gel electrophoresis
PBS	Phosphate-buffered saline
PI	Propidium iodide
PI3K	Phosphoinositide 3-kinase
QA	Quinic acid
RI	Retention index
SDS	Sodium dodecyl sulfate
TBS-T	TRIS-buffered saline with Tween 20
TCA	Trichloroacetic acid
TIC	Total ion current
TMS	Trimethylsilyl
TNF-α	Tumor necrosis factor-alpha
TRIS	Tris(hydroxymethyl)aminomethane
UFA	Unsaturated fatty acids
UPW	Ultra-pure water
