# Supplementary material for: LC-PDA-MS and GC-MS Analysis of Scorzonera hispanica Seeds and Their Effects on Human Breast Cancer Cell Lines
Source: Int J Mol Sci. 2022 Sep 30;23(19):11584. doi: 10.3390/ijms231911584 (PMC9569732; doi:10.3390/ijms231911584)
Supplement: Supplementary file 1 [file ijms-23-11584-s001.zip › ijms-1908111-FS1 and S2 supplementary.pdf]

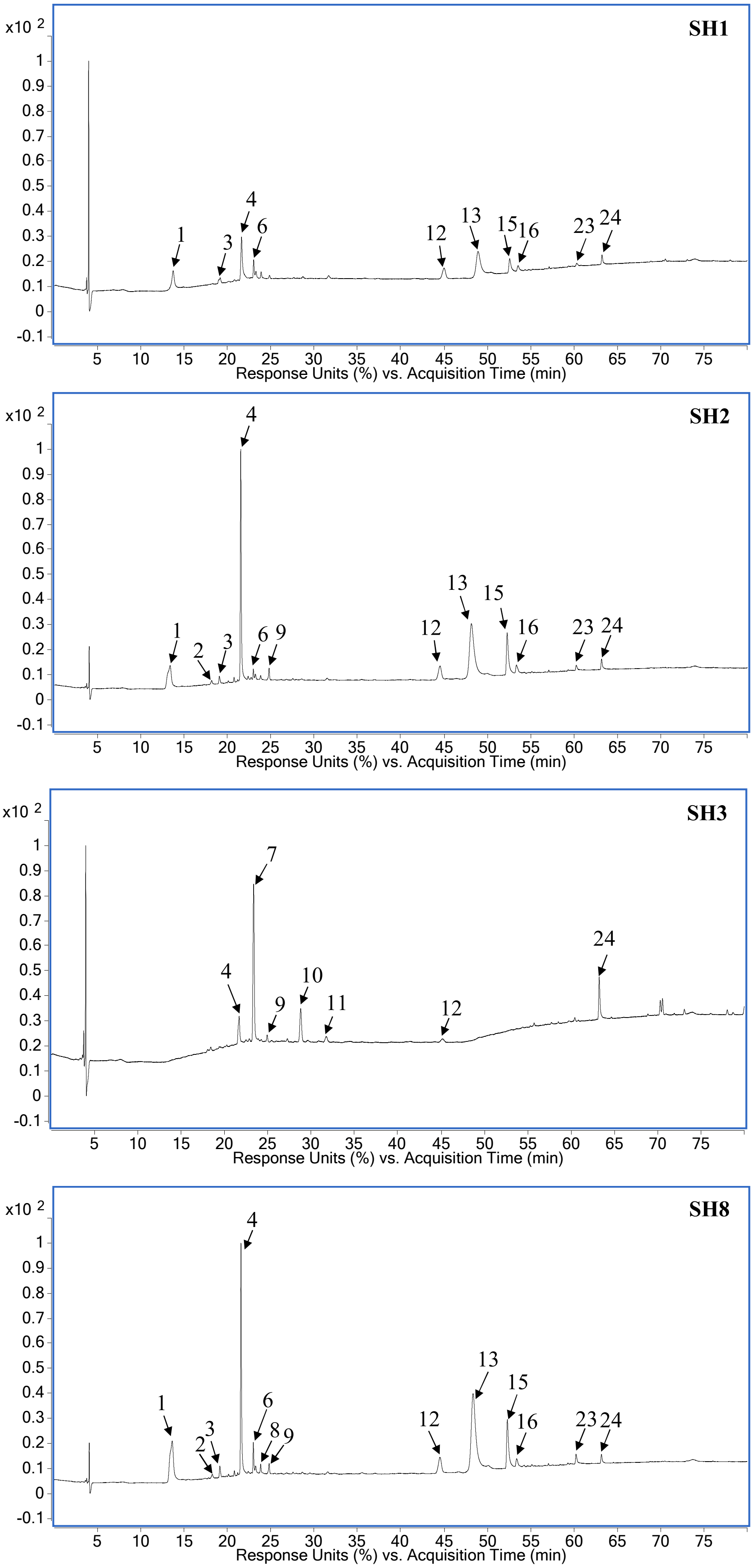

Figure S1. UV–VIS chromatogram of SH1-3 and SH8 extracts obtained by HPLC-PDA-MS (280 nm).

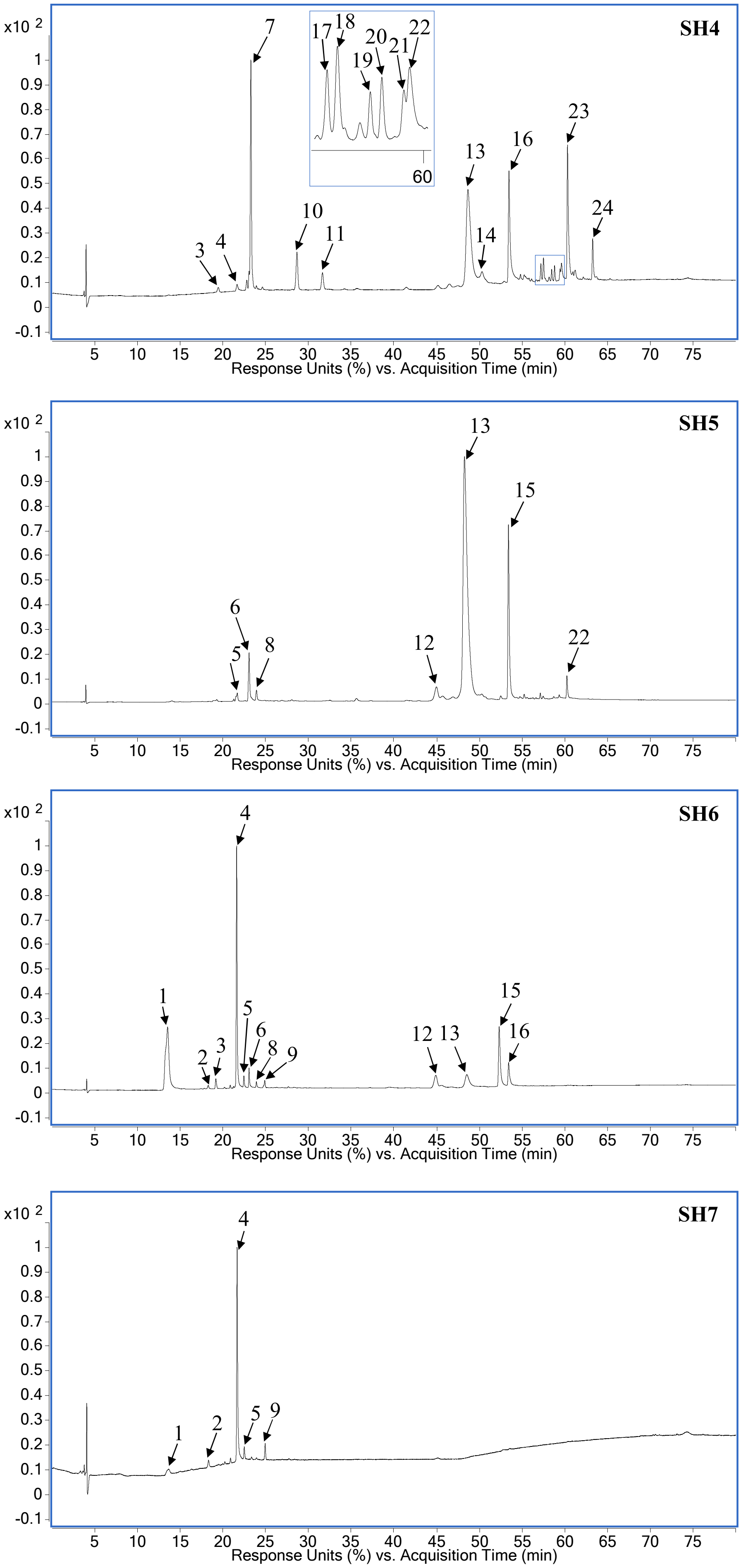

Figure S2. UV–VIS chromatogram of SH4-6 fractions and SH7 residue obtained by HPLC-PDA-MS (280 nm).
