# Supplementary material for: LC-PDA-MS and GC-MS Analysis of Scorzonera hispanica Seeds and Their Effects on Human Breast Cancer Cell Lines
Source: Int J Mol Sci. 2022 Sep 30;23(19):11584. doi: 10.3390/ijms231911584 (PMC9569732; doi:10.3390/ijms231911584)
Supplement: Supplementary file 1 [file ijms-23-11584-s001.zip › IJMS-1908111-FS3-S7 supplementary.pdf]

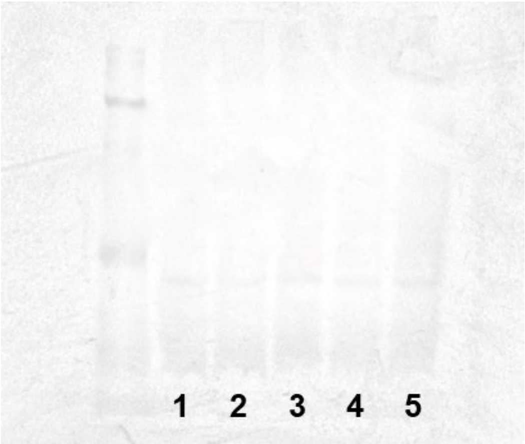

Figure S3. Original Western blotting membrane used to evaluate the expression of BCL-2 in MCF-7 breast cancer cells. 1) Control; 2) SH1 800 µg/mL; 3) SH4 800 µg/mL; 4) SH11 400 µg/mL; 5) Cisplatin 100 µM.

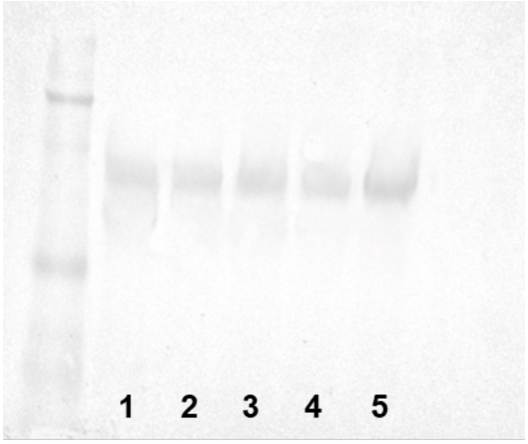

Figure S4. Original Western blotting membrane used to evaluate the expression of ATG5 in MCF-7 breast cancer cells. 1) Control; 2) SH1 800 µg/mL; 3) SH4 800 µg/mL; 4) SH11 400 µg/mL; 5) Cisplatin 100 µM.

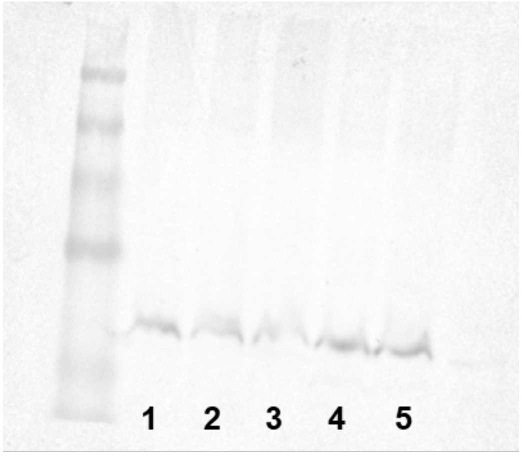

Figure S5. Original Western blotting membrane used to evaluate the expression of BAX in MCF-7 breast cancer cells. 1) Control; 2) SH1 800 µg/mL; 3) SH4 800 µg/mL; 4) SH11 400 µg/mL; 5) Cisplatin 100 µM.

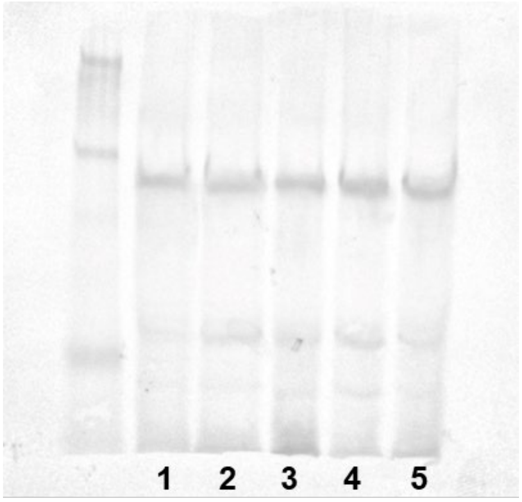

Figure S6. Original Western blotting membrane used to evaluate the expression of LC3B in MCF-7 breast cancer cells. 1) Control; 2) SH1 800 µg/mL; 3) SH4 800 µg/mL; 4) SH11 400 µg/mL; 5) Cisplatin 100 µM.

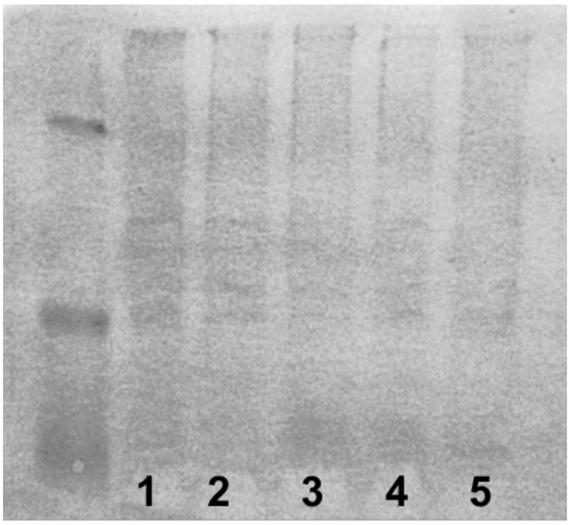

Figure S7. Original Western blotting membrane used to evaluate the expression of p-FAK in MCF-7 breast cancer cells. 1) Control; 2) SH1 800 µg/mL; 3) SH4 800 µg/mL; 4) SH11 400 µg/mL; 5) Cisplatin 100 µM.
